# Open ligamentous complex disruption of the lateral ankle without dislocation or fracture: Two case reports

**DOI:** 10.1097/MD.0000000000017447

**Published:** 2019-10-11

**Authors:** Dong-Il Chun, Jahyung Kim, Sung Hun Won, Young Yi, Yong-Been Kim, Jaeho Cho

**Affiliations:** a Department of Orthopedic Surgery, Bone and Joint center, Soonchunhyang University Seoul Hospital,; b Department of Orthopedic Surgery, Seoul Hospital, Soonchunhyang University Seoul Hospital,; c Department of Orthopedic Surgery, Seoul Paik Hospital, Inje University College of Medicine, Seoul,; d Department of Orthopaedic Surgery, Chuncheon Sacred Heart Hospital, Hallym University College of Medicine, Chuncheon, Republic of Korea.

**Keywords:** ankle, basketball, dislocation, fracture, lateral ligament complex

## Abstract

**Introduction:**

Lateral ligamentous complex injury without fracture or dislocation is rare in the literature. Due to the rare injury, it is not clear yet about the proper treatment. This case report suggests a specific diagnosis of this injury as well as an appropriate surgical method.

**Patient concerns:**

In one-month period of time, 2 male soldiers participating in recreational basketball game presented with open wound on the lateral aspect of ankle without associated dislocation or fracture when they attempted to rebound the ball which consequently made them land on another player's foot.

**Diagnosis:**

Total rupture of the lateral ligament complex with open wound was found without any associated fracture or dislocation.

**Interventions:**

Open repair of the ruptured ligaments and capsule was performed.

**Outcomes:**

Patients returned to his own job's duty with none to minimal limitation in sport and activities of daily living at 9 to 10 months after the injury.

**Conclusion:**

Although open disruption of the lateral ligamentous complex without fracture or dislocation is rare, an adequate assessment and prompt surgical repair led to satisfactory outcome.

## 1 Introduction

The lateral ankle sprain is the most common injury in sports.^[1]^ Especially in basketball, ankle sprains account for 45% of all injuries^[1]^. Majority of these injuries are often limited to anterior talofibular ligament and almost always occur without open wound. On the other hand, ligament injury with ankle dislocation may occur in open manner with the form of laceration of soft tissue that covers the malleoli.^[2,3]^ In summary, open rupture of the lateral ligaments of the ankle without dislocation or fracture is a rare injury, with few cases reported in the literature. In this case report, we cover 2 soldiers’ open rupture of the lateral ligaments of the ankle without dislocation and fracture participating in the recreational basketball.

## 2 Case reports

### 2.1 Case 1

**Figure 1:**
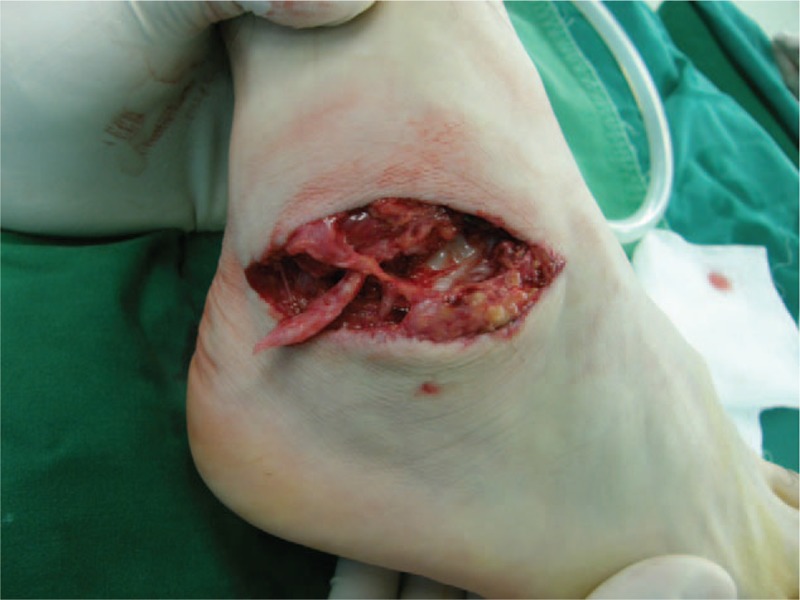
Photograph showing the open rupture of the lateral ligaments of the ankle at presentation.

**Figure 2:**
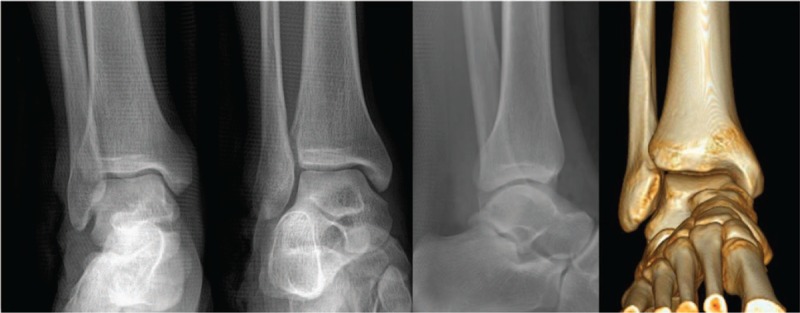
Ankle is not fractured or dislocated.

A 20-year-old male soldier participating in recreational basketball got injury on his right ankle after attempting rebound. The mechanism of injury was described as an inversion after landing on another player's foot. He described no history of previous ankle injuries. On examination there was 8 cm sized jagged laceration over the dorso-lateral aspect of right ankle (Fig. 1). The lateral ligaments and torn capsule were visible through the wound. There was no gross deformity of the ankle. Sensory and motor examination was normal and dorsalis pedis and posterior tibial pulses were intact. Plain radiographs and 3-dimensional CT (3DCT) indicated no fracture or dislocation (Fig. 2). Surgical management was decided. The previous open wound was extended for better exposure. The anterior talofibular ligament (ATFL), calcaneofibular ligament (CFL), and anterior portion of posterior talofibular ligament (PTFL) were ruptured. The anteroinferior tibiofibular ligament (AITFL) and peroneal tendons were in their normal shape. Inspection of the ankle joint revealed no evidence of cartilage injury.

**Figure 3:**
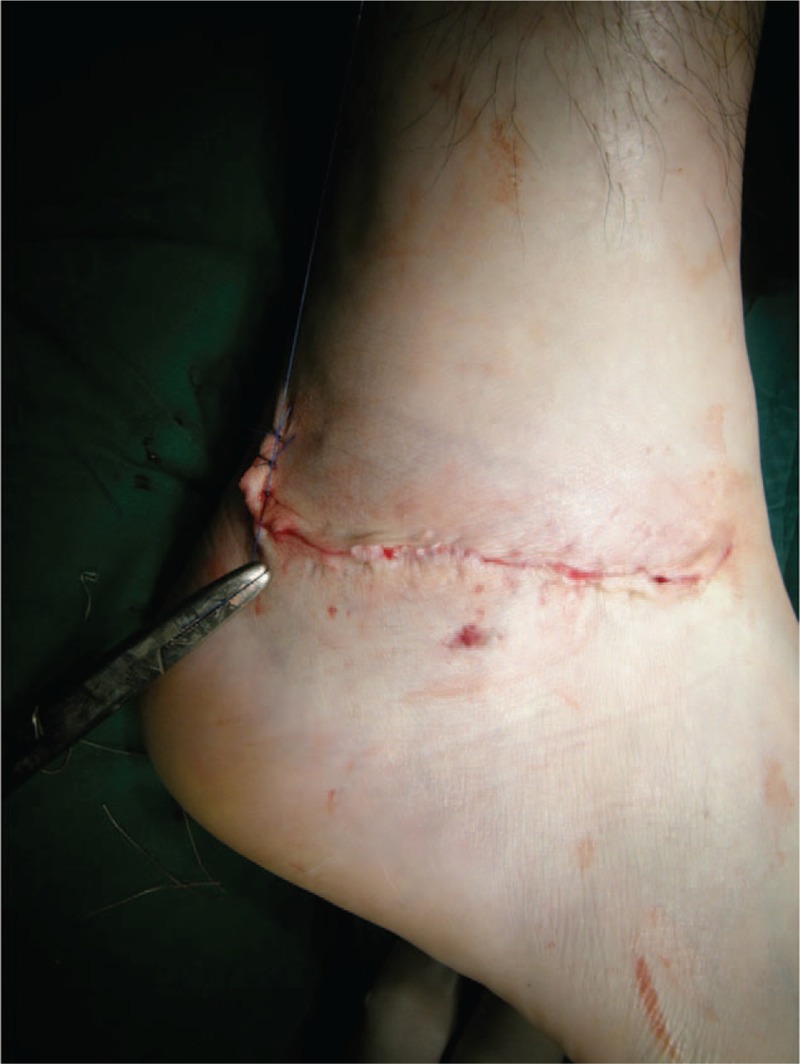
The wound was closed without tension after repair lateral ligaments.

The 2–0 ethibond sutures were used to repair the ligaments and 2–0 absorbable sutures were added to repair the torn capsule. The wound was closed without tension (Fig. 3). The ankle was immobilized in short leg-cast for 6 weeks. The wound healed without complications. After cast removal, rehabilitation exercises including joint motion and muscle strengthening were followed for 4 weeks. By 9 months after injury, the patient returned to his own job's duty and reported no limitation in sport and activities of daily living.

### 2.2 Case 2

**Figure 4:**
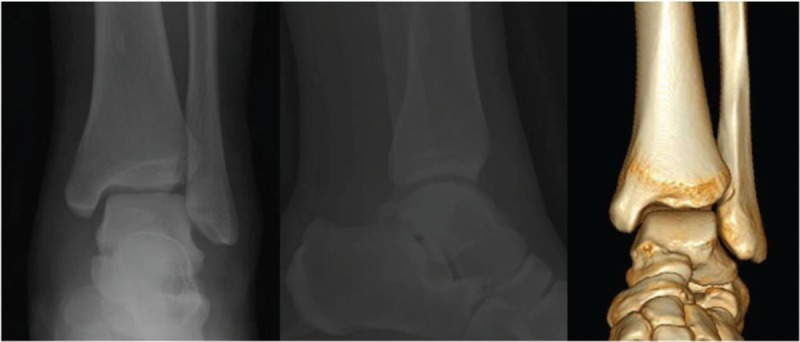
Ankle is not fractured or dislocated.

A 21-year-old male soldier participating in the recreational basketball was injured his left ankle. The mechanism of injury was the same as that in case 1. On examination there was 4 cm sized laceration over the dorso-lateral aspect of left ankle. The lateral ligaments and torn capsule were visible through the wound. There was no gross deformity of the ankle. Sensory and motor examination was normal and pulse surrounding ankle was intact. Plain radiographs & 3DCT indicated no fracture or dislocation (Fig. 4). Because the authors experienced similar injury (as described in case 1) just three weeks earlier, prompt surgical repair was decided. The ATFL and anterior portion of CFL were found to be ruptured under surgical exploration. The AITFL and peroneal tendons were intact. No evidence of cartilage injury was found.

**Figure 5:**
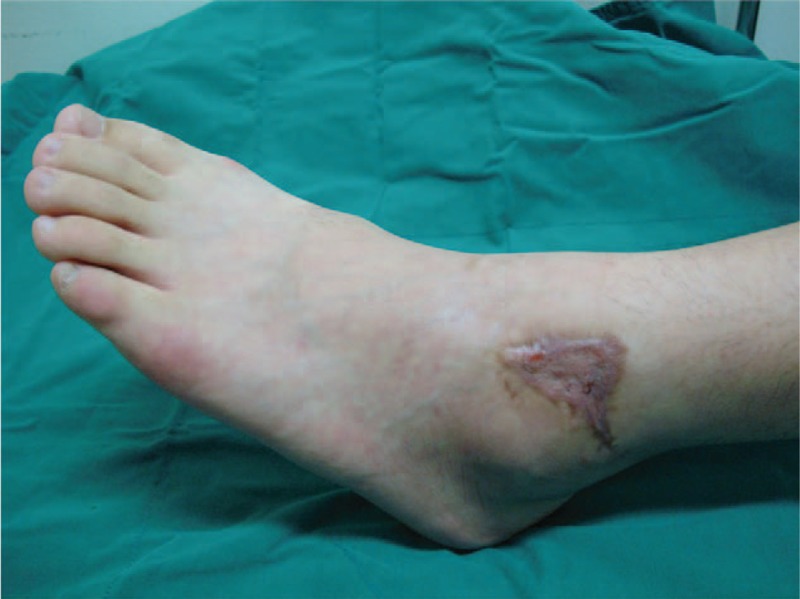
Wound healed completely after split-thickness skin graft.

Surgical repair attempted in the same manner as case 1. Two weeks after operation, wound infection and skin necrosis occurred. Vacuum assisted closure therapy after wound debridement was applied for 10 consecutive days. Wound was satisfactorily granulated and split-thickness skin graft was applied additionally (Fig. 5). By 10 months, clinical examination confirmed a full pain-free range of movement and he experienced some degree of discomfort in the ankle after sport activity but no disabling pain. He has no pain in his daily life.

The present study was approved by the Institutional Review Board and Human Research Ethics Committee of Hallym University Chuncheon Sacred Heart Hospital (No. 2019-02-012). Consent forms for publication of the cases were obtained from the patients.

## 3 Discussion

Acute lateral ankle injuries usually occur when the weight of the body lands on the plantar-flexed, internally rotated ankle, leading to diminished bony stability of the ankle mortise.^[4]^ Such mechanism causes decrease in the talar weight bearing surface, interrupts the full loading on the articular surface, and the ligaments consequently absorb added stress.^[2]^ A typical example happens in basketball when ankle becomes inverted after landing on another player's foot.

The ATFL is the first of lateral ligaments to fail because of an inversion injury, next are the CFL and the PTFL.^[5]^ Partial or complete rupture of the ATFL is most common in lateral ankle sprain, whereas rupture of all 3 ligaments is considered to be the most severe injury.^[5]^ Several authors reported that lateral ligaments tears usually occurred with event of ankle dislocation.^[3,5,6]^ Furthermore, most of the ankle dislocation occur with fracture due to the strength of the collateral ligaments and the capsular reinforcements of the mortise capsule, which may exceed that of the malleolus and hence in high energy trauma, resulting in subsequent bone injury.^[7]^

In our cases, extreme inversion force applied to the ankle caused maximal tension of the skin overlying the lateral malleolus and ultimately resulted in laceration. The degree of lateral ligament rupture depends upon the severity of its injury. Although the possibility of transient subluxation of the ankle joint at the time of injury cannot be excluded, our patients reported that there had been no gross deformity and no ankle manipulation had been carried out before they presented to the emergency room.

Open ligamentous disruption without associated fracture or dislocation is rarely reported. In our knowledge, three previous reports dealing with this subject have been published. Bryant et al^[8]^ reported 2 cases of patients who experienced inversion injury during rock climbing. They suggested that the rock-climbing shoe can cause increased stress on lateral ligament and capsule when the ankle is damaged. Carter et al^[9]^ reported similar injury to 25 years old basketball player, which was successfully treated with immobilization and functional rehabilitation.

There is no standard treatment protocol to this type of injury due to its rare occurrence. As described above, Carter et al^[9]^ applied external fixator for immobilization due to extensive destruction and stretching of the capsule which was not suitable to repair. On the other hand, Bryant et al and Thompson and Muhammad^[10]^ obtained satisfactory outcome after performing surgical repair to such injury as in our cases. Surgical repair has benefits and drawbacks. First, the extent of the injury can be underestimated without surgical exploration due to the normal findings on radiologic examinations. Second, surgical management of lateral ligament complex ruptures has been known to satisfactorily restore function and lead to a high rate of return to pre-injury level of activity.^[11]^ However, skin problems can be one of the matters as described in case 2 which required skin graft. Therefore, surgical repair without excessive tension on the ligament and skin should be considered.

## 4 Conclusions

Injuries with open disruption of the lateral ligamentous complex without fracture or dislocation are not common. Therefore, an adequate assessment should always be carried out, with high level of suspicion on the extent of injury when the orthopedic surgeons face the patients with open wound on lateral malleolus describing history of ankle inversion accident. After a precise diagnosis, prompt surgical repair of the injured ligaments and skin without excessive tension may lead to satisfactory outcome.

## Author contributions

**Conceptualization:** Dong-Il Chun, Sung Hun Won, Young Yi, Jaeho Cho.

**Data curation:** Yong-Been Kim.

**Writing – original draft:** Jahyung Kim, Jaeho Cho.

**Writing – review & editing:** Dong-Il Chun, Jaeho Cho.

Jaeho Cho orcid: 0000-0001-8680-4680.
